# Effect of Ca(OH)_2_ Addition on the Engineering Properties of Sodium Sulfate Activated Slag

**DOI:** 10.3390/ma14154266

**Published:** 2021-07-30

**Authors:** Xiaodi Dai, Serdar Aydın, Mert Yücel Yardımcı, Karel Lesage, Geert De Schutter

**Affiliations:** 1 Magnel-Vandepitte Laboratory, Department of Structural Engineering and Building Materials, Ghent University, 9052 Ghent, Belgium; Xiaodi.Dai@UGent.be (X.D.); MertYucel.Yardimci@UGent.be (M.Y.Y.); Karel.Lesage@ugent.be (K.L.); 2 Department of Civil Engineering, Dokuz Eylül University, Izmir 35160, Turkey; 3 Department of Civil Engineering, Istanbul Okan University, Istanbul 34959, Turkey

**Keywords:** ground granulated blast furnace slag, sodium sulfate, calcium hydroxide, alkali-activated cements

## Abstract

Alkali-activated slag is considered as a sustainable construction material due to its environmentally friendly nature. To further promote the sustainable nature of alkali-activated slag, a sodium sulfate activator is suggested to be used since it can be obtained naturally and generates lower greenhouse gas emissions. However, the mixtures activated by sodium sulfate exhibit low early strength and very long setting times. This study investigates the effects of calcium hydroxide (Ca(OH)_2_) addition on some engineering properties such as rheology, setting time, mechanical properties, porosity, and microstructure of sodium sulfate activated ground granulated blast furnace slag (GGBFS). Furthermore, the changes of chemical groups in reaction products and phase identification have been evaluated by Fourier transform infrared spectroscopy (FTIR) and X-ray diffraction. Test results showed that Ca(OH)_2_ addition can substantially increase the reaction rate and the compressive strength at early ages. In addition, the very long setting times of the sodium sulfate-activated mixtures were shortened by the addition of Ca(OH)_2_. SEM analysis confirmed that the incorporation of excessive amounts of Ca(OH)_2_ could lead to a less well-packed microstructure although the reaction degree of GGBFS remained the same at later ages as compared to the sodium sulfate mixture. It was also revealed that in case of the Ca(OH)_2_ addition into sodium sulfate activator, the main reaction products are chain-structured C-A-S-H gels and ettringite.

## 1. Introduction

Alkali-activated cements (AACs) or geopolymers produced from the reaction of an alkali metal source (solid or dissolved) with a solid aluminosilicate powder [1] are more environmentally-friendly and require less energy compared to conventional ordinary Portland cement (OPC) based cementitious materials [2]. AACs, as one of the most promising alternatives to the OPC, have equivalent or better performance than conventional cementitious binders. It has been stated that AACs can have less greenhouse gas emission (CO_2_), superior mechanical properties and better durability performance against high temperature, acid and sulfate attacks when compared to several types of existing OPC-based concrete [3,4,5,6,7,8]. Among all AACs, ground granulated blast furnace slag (GGBFS) activated by sodium silicate and/or sodium hydroxide is the most intensively studied since it provides the best formulation for high strength and other advantageous properties. However, both sodium silicate and sodium hydroxide do not exist naturally and must be obtained through an energy-intensive manufacturing process. This is particularly true for sodium silicate which is made by melting sand and sodium carbonate at 1350–1450 °C and then dissolving it in an autoclave at 140–160 °C under appropriate steam pressure [1]. Consequently, sodium silicate and sodium hydroxide activation may not be the best solution for achieving a sustainable cementitious system.

Sodium sulfate (Na_2_SO_4_) is suggested as a cheaper, cleaner, less harmful and more environmentally friendly alternative activator to sodium silicate and sodium hydroxide for the activation of slag [9]. This is due to sodium sulfate being found naturally as thenardite (anhydrous Na_2_SO_4_) and mirabilite (Na_2_SO_4_·10H_2_O) [10]. As reported by Mobasher et al. [10], the main reaction products in Na_2_SO_4_ activation are calcium-aluminosilicate-hydrate (C-A-S-H) type phases with a low Ca/Si ratio, providing the main contribution to the strength of the AAC and ettringite (3CaO·Al_2_O_3_·3CaSO_4_·32H_2_O) as a secondary reaction product [11]. In this activation, ettringite is stable even at high alkalinity due to the high sulfate contents of the system [11,12]. The Na_2_SO_4_ sodium sulfate activated slag has been considered as a possible binder for coping with certain radioactive wastes containing reactive metals due to its lower pH, heat of hydration and lower free water content [12]. However, there have been limited studies on the activation of slag with sodium sulfate as compared to sodium silicate and sodium hydroxide. Possibly, this is owing to the sodium sulfate-activated slag mixtures exhibiting low early strength [9,10,13]. A higher early strength can be obtained in these systems by grinding the slag to a finer particle size, curing at higher temperatures, or using a combined chemical activator to obtain a higher early strength in these systems. Rashad et al. [9] pointed out that increasing the slag fineness is a more effective method than increasing the concentration of Na_2_SO_4_ for optimizing early and long-term strength of Na_2_SO_4_ activated slag mixtures. Fu et al. [13] also reported that the addition of Na_2_SO_4_ in conjunction with Portland cement improved slag hydration through a complex process. Gijbels et al. [14] also investigated the effect of the sodium hydroxide content on alkali/sulfate-activated binders from 90 wt% GGBFS and 10 wt% phosphogypsum, and found that a high ettringite content can increase the compressive strength. Zhang et al. [15] pointed out that using ultra-fine GGBFS improves the mechanical performance of Na_2_SO_4_ activated slag mixtures at ambient temperature.

To date, the studies concerning rheological and mechanical properties of sodium sulfate activated GGBFS mixtures are very limited. Although sodium sulfate-activated GGBFS mixtures can significantly reduce the carbon footprint, their drawbacks of lower compressive strength, longer setting time and higher porosity, severely limit their applications. This study aims to investigate the influence of Ca(OH)_2_ addition on the engineering properties of sodium sulfate activated slag such as rheological properties using a rheometer, mechanical properties using compressive and flexural strength tests, porosity using mercury intrusion porosimetry (MIP), microstructure using a scanning electron microscope/backscattered electron (BSE) imaging, chemical groups in reaction products using Fourier transform infrared spectroscopy (FTIR) and phase identification using X-ray diffraction (XRD). Test results showed that the engineering properties of the Na_2_SO_4_ activated slag cements can be significantly improved by the addition of Ca(OH)_2_.

## 2. Materials and Methods

### 2.1. Materials and Sample Preparation

Ground granulated blast furnace slag (GGBFS) was used as the precursor material in this study. The chemical composition of GGBFS was determined by X-ray fluorescence spectrometry (XRF), and is shown in Table 1.

**Figure 1:**
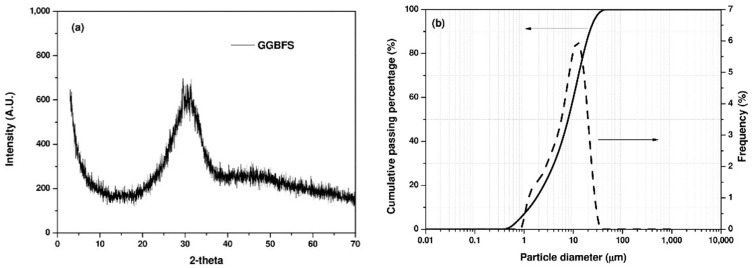
(**a**) X-ray diffraction pattern and (**b**) particle size distribution of GGBFS.

The X-ray diffraction (XRD) pattern and particle size distribution of GGBFS, determined by a powder diffractometer and laser diffraction, respectively, are shown in Figure 1.

**Figure 2:**
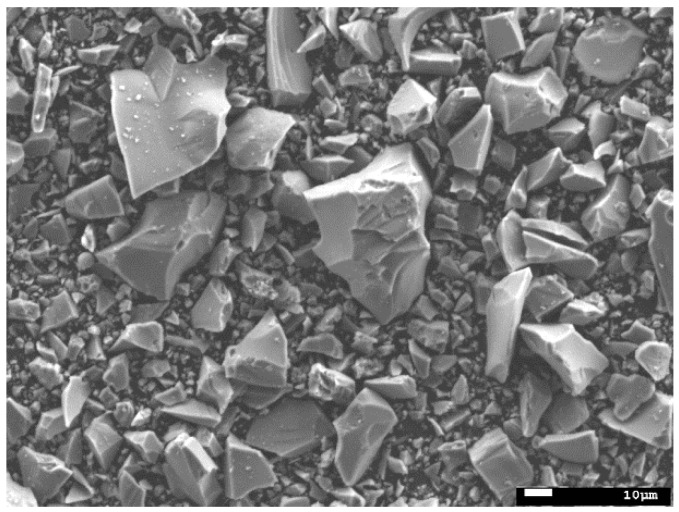
SEM image of GGBFS particles.

The GGBFS shows a broad diffraction peak between 20° and 35° 2θ because of its amorphous components, and no crystalline phases were observed. The volume–mean particle size (d_50_) of GGBFS was around 9 μm. The morphology of GGBFS particles observed by scanning electron microscope (SEM) is shown in Figure 2. As can be seen in Figure 2, GGBFS shows irregular shapes with high angularity.

The mixture compositions are shown in Table 2. The mixtures are indicated with the abbreviation of their activators (e.g., SS is for sodium sulfate) and the weight percent of the Na_2_O from the activator with respect to the GGBFS amount in the mixture. For instance, the mixture notation of SS5% for Mix 1 shows that the activator of Mix 1 consists of sodium sulfate (SS) and the Na_2_O content from the SS are 5%. The mixture notation of SS5% + CH0.5% for Mix 2 shows that the activator of Mix 2 consists of sodium sulfate (SS) and calcium hydroxide (CH), and the Na_2_O content from the SS is 5% while CH is 0.5% of the mass of the GGBFS. The water to solid binder ratio of 0.42 was kept constant in all mixtures.

Sodium sulfate (98% purity) and calcium hydroxide (slaked lime) were used as the activators. Activator solutions were prepared by mixing the sodium sulfate and calcium hydroxide with water one day before the preparation of the paste or mortar mixtures. To ensure the complete dissolution of the sodium sulfate, the activators containing sodium sulfate were kept at 40 ± 1 °C for one day, and cooled down to 20 °C before use. For the preparation of the paste samples, the activator solution was poured into a Hobart mixer bowl first, then the GGBFS was added and mixed at low (140 ± 5 rpm) and high (285 ± 5 rpm) speeds for 90 s each, respectively. Afterward, the pastes were cast into the 40 × 40 × 160 mm steel molds and vibrated for 1 min to remove the air bubbles as much as possible for full compaction. All the measurements were done at a temperature of 20 °C.

### 2.2. Test Methods

**Figure 3:**
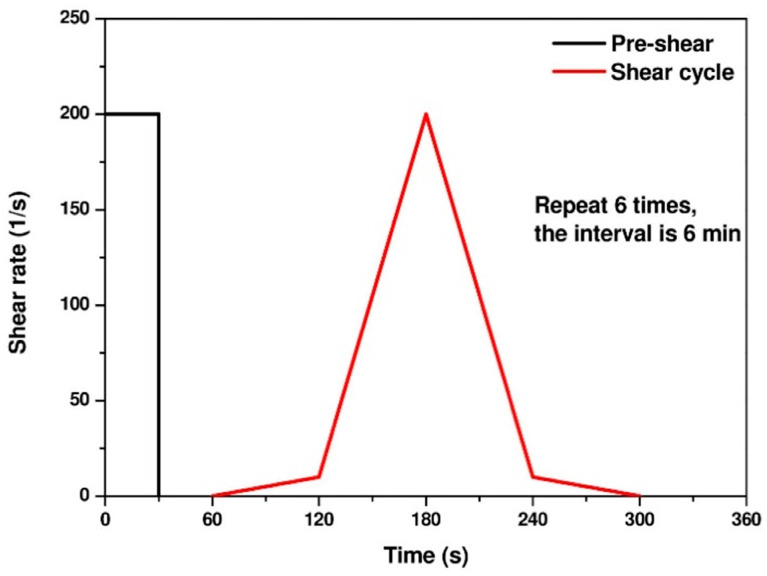
Shear protocol.

**Figure 4:**
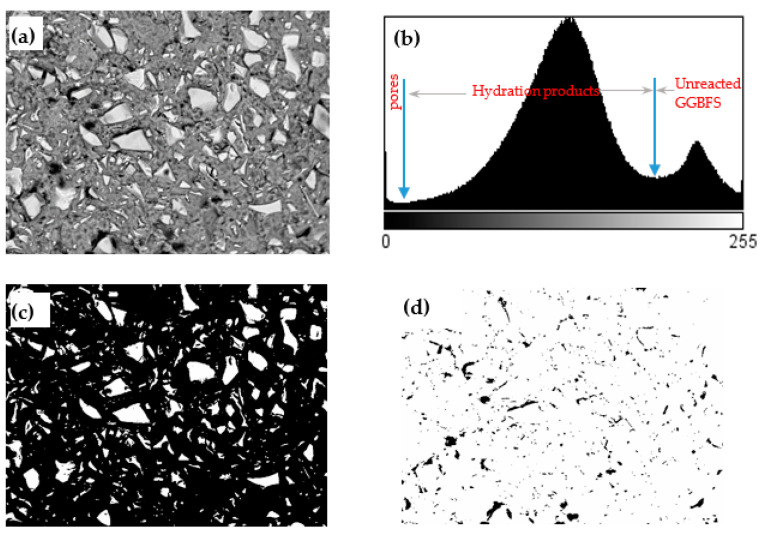
(**a**) Filtered backscattering electron (BSE) image by bilateral filter, (**b**) grayscale histogram (**c**) unreacted GGBFS, (**d**) cracks or pores obtained by applying threshold on the filtered BSE image.

- Rheological tests: The flow curves of the pastes were obtained by the shear protocol presented in Figure 3 for determining viscosity and yield stress. The rheological parameters were determined by considering the descending part of the curves. The downward curve of all mixtures followed a modified Bingham model (Equation (1)) and was used to determine the dynamic yield stress and plastic viscosity of the mixtures.
  (1) $$\tau =\tau_{0}+\mu \dot{\gamma }+c{\dot{\gamma }}^{2}$$
  where, τ is the shear stress in Pa, $\tau_{0}$ is the yield stress in Pa, μ is the plastic viscosity in Pa·s and $\dot{\gamma }$ is the shear rate in 1/s, c is the second order term (Pa·s^2^). All rheological tests were carried out on three fresh samples.
- Setting times: The initial and the final setting times were determined by an automatic Vicat apparatus according to EN196-3:2005 [16] on paste samples. The initial setting time was determined by the elapsed time from the first contact of the slag particles with the alkaline activator to the time at which the distance between the needle and the base-plate was (6 ± 3) mm. The final setting time was determined by the time at which the needle can penetrate only 0.5 mm from the surface.
- Mechanical properties: The compressive and flexural strength of the mixtures were measured on mortar samples. Standard CEN sand was used as aggregate, and an aggregate to binder ratio of three (by mass) was kept constant for all mortar mixtures. The fresh mortar specimens were wrapped in plastic foil and stored in the moisture room for 24 h at 20 °C and 90% RH. The specimens were stored in the same environmental conditions after demolding until the mechanical tests at 2, 7, and 28 days. The compressive strength of the specimens was determined on the two broken portions of prisms after the flexural test according to EN 1015-11 [17]. At least three samples for each mixture were tested to achieve reproducibility.
- Mercury intrusion porosimetry (MIP): At the testing ages (2, 7, and 28 d), the paste samples were crushed into small pieces with dimensions of around 1 cm^3^, and then the small pieces of the samples were immersed in isopropanol for at least one week to stop the reaction of the slag and dried in a 40 °C oven for 1 h. Then the dried samples were stored in a low vacuum desiccator before analysis. A Pascal 440 mercury porosimeter with a maximum load capacity of 420 MPa was used in the MIP test. However, the maximum pressure was limited to 200 MPa in order to avoid cracks induced by the mercury pressure [18]. The adopted mercury surface tension and contact angle between the mercury and the solid surface were 0.482 N/m and 142°, respectively.
- Scanning electron microscopy (SEM) and image analysis (IA): Paste samples at the age of 28 days were immersed into epoxy and then polished up to 0.25 μm surface fineness using diamond paste. Afterward, the polished samples were observed by an SEM in backscattering electron (BSE) mode at an acceleration voltage of 15.0 kV under low vacuum. The magnification of each image was 500×. A representative BSE image and the analytical procedure for calculating the reaction degree from BSE images are illustrated in Figure 4. The discrimination between hydrated/anhydrous regions using the original BSE image was not easy, since no distinctive peaks according to gray level are observable from the gray-level histogram. The Bilateral filter available in the ImageJ software (https://imagej.nih.gov/ij/ accessed on 20 January 2021) was used to make the BSE images smoother and keep the particle edges in BSE images. The gray-scale histogram (Figure 4b) was obtained from the cropped/filtered image. As can be seen from Figure 4b, there were two distinct regions allowing the determination of the thresholds for quantifying the areas corresponding to unreacted GGBFS and hydrated phases, as well as cracks or pores. By applying an appropriate threshold value (Figure 4b), the areas corresponding to the unreacted GGBFS was obtained as shown in Figure 4c. The first principle of stereology (or the Delesse Principe [19]), states that a determination of the area fraction of a phase in a random section, is an unbiased estimator of the volume fraction of this phase. As such, the degree of reaction of GGBFS can be estimated as follows:
  (2) $$\alpha \;\left(t\right)=\left(1-\frac{V_{t}}{V_{i}}\right)\times 100\%≅\;\left(1-\frac{A_{t}}{A_{i}}\right)\times 100\%$$
  where α (t) is the degree of reaction of GGBFS at age t, $V_{t}$ and $V_{i}$ are the volume fraction of unreacted slag relative to the total volume of mixture at given curing time and at the initial state, respectively, and $A_{t}$ and $A_{i}$ are the area fraction of unreacted slag relative to the total cross section of mixture at a given curing time and at the initial state, respectively, as obtained by the BSE image analysis. The initial volume fraction of the GGBFS was calculated as 45.16% and 45.35% for Mix 1 and 4, respectively. It has been reported that an image analysis based on 12 or more BSE images can enable a 95% degree of confidence [20]. Consequently, a total number of 20 images on randomly selected locations for each sample were used in image analysis.
- Fourier transform infrared (FTIR) spectroscopy: The samples were taken from the hardened paste samples at an age of 28 days. Following the RILEM TC-238 [21] methodology, after crushing the hardened pieces to a size of 125 μm to 1 mm, 3 g of the powder was mixed with 100 mL isopropanol for 15 min. The suspension was filtered and rinsed with isopropanol and diethyl ether before drying at 40 °C for 8 min. Then the dried samples were stored in a low vacuum desiccator prior to analysis. KBr pellets were prepared by mixing 1 mg of sample and 100 mg of KBr. The FTIR tests were conducted on a Perkin Elmer spectrum BX FT-IR system, in the frequency range of 400–4000 cm^−1^ with a 4 cm^−1^ resolution.
- X-ray diffraction (XRD): The sample preparation procedure was the same as those for FTIR. The XRD measurements were conducted on a Rigaku D/Max-2200/PC X-ray diffractometer with CuKα radiation (λ = 0.1542 nm) at 40 kV and 36 mA, scanning from 5° to 70° 2θ with a 0.02° step size.
- Isothermal calorimetry: The heat of the hydration of the pastes was measured using a TAM air calorimeter with eight channels. Immediately after mixing the activator solution with GGBFS for 3 min, 14 g of paste sample was poured into a glass ampoule bottle and then the sealed bottle was placed into the calorimeter. The calorimetric measurements were performed at 20 ± 0.02 °C for 7 d.

## 3. Results and Discussions

### 3.1. Isothermal Calorimetry of AAC Pastes

**Figure 5:**
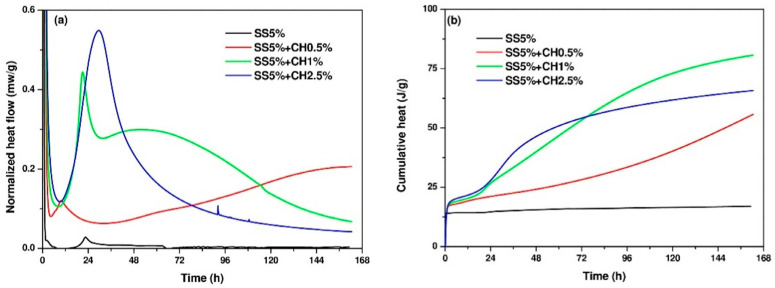
(**a**) Heat evolution and (**b**) cumulative heat of AAC pastes.

Figure 5 presents the heat release and cumulative heat of AAC pastes. As can be seen from Figure 5, for sample SS5% without the addition of Ca(OH)_2_, only a very low exothermic peak could be observed at around 22–23 h, but it was basically negligible to the total heat release. As such, no exothermic reactions took place during first 7 days. This agrees with the zero compressive strength as presented in Section 3.4. The addition of CH significantly improved the reaction rate. When the addition of CH was 1%, the heat release curve presented a different behavior compared to the other AAC mixtures with distinct periods of acceleration and deceleration. After induction period, the heat release curve showed a small exothermic peak, followed by a plateau with rate of around 0.25 mw/g for nearly 48 h, then smoothly decreased. The sample SS5% + CH1% also exhibited the highest cumulative heat release compared to the other two mixtures after 7 days.

### 3.2. Flow Curves of AAC Pastes

**Figure 6:**
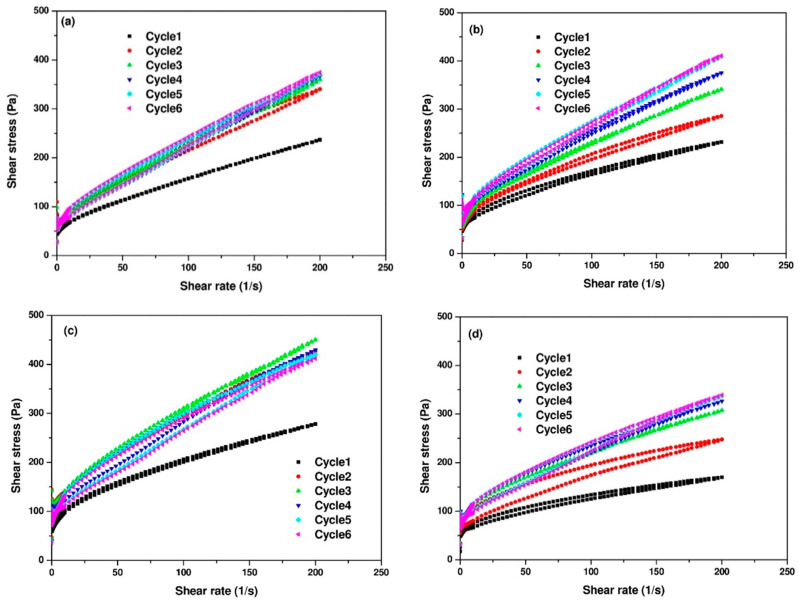
Flow curves of AAC pastes (**a**) SS5%, (**b**) SS5% + CH0.5%, (**c**) SS5% + CH1%, (**d**) SS5% + CH2.5%.

Figure 6 presents the flow curves of the AAC paste mixtures with different doses of CH. Yield stress and plastic viscosity of the AAC paste mixtures are obtained from the descending part of each shear cycle at ages of 10, 20, 30, 40, 50, and 60 min.

**Figure 7:**
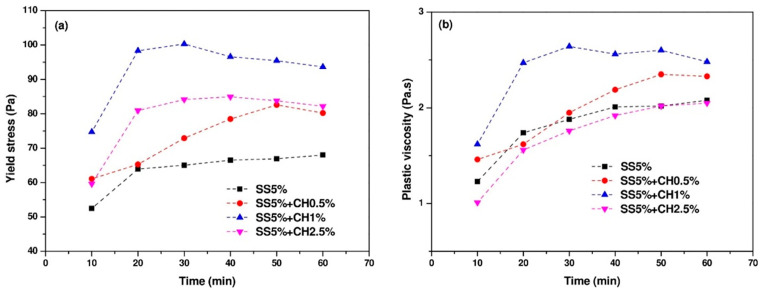
The evolution of (**a**) yield stress and (**b**) plastic viscosity.

As can be seen in Figure 7a, mixture SS5% showed the lowest yield stress during the measurement time, while with addition of CH, the yield stress increased rapidly. In particular for the mixture with the addition of 1% CH, the yield stress reached 75 Pa at an age of 10 min, with time elapsing, the yield stress fluctuated around 95 Pa. This shows that CH addition can introduce more solid formation into the suspension, thereby leading to higher yield stress. Additionally, the plastic viscosity of AAC pastes also increased with the addition of CH as shown in Figure 7b. Similar to the trend of the evolution of yield stress, the addition of 1% CH resulted in a higher plastic viscosity. However, when excessive CH was added at a dosage of 2.5%, the plastic viscosity was the lowest as compared to other mixtures.

### 3.3. Initial and Final Setting Times of AAC Pastes

The initial and final setting times of AAC pastes are shown in Table 3. Setting times with different activator solutions varied in a wide range. The sodium sulfate mixture with Na_2_O content of 5% (Mix 1) showed very long initial and final setting times reaching 2940 and 3960 min, respectively. Correspondingly, this mixture did not present any measurable compressive strength up to 7 days, as presented in Section 3.3. Besides the very low reaction product formation rate contributing to the very long setting time of this mixture, reasons for the slow setting of this mixture could also be some physical phenomena: air bubble migration to bleeding, workability loss due to intake of the solution by initial chemical reactions and densification of the internal structure by settling of the particles due to gravity, causing a mechanical bounding between particles [22]. Initial and final setting times of sodium sulfate mixtures significantly shortened with the incorporation of CH. Mix 2 and Mix 4 had similar initial setting times, while Mix 3 showed relatively shorter initial and final setting times than Mix 2 and Mix 4, reflecting that an appropriate dosage of CH may contribute to shorter setting time.

### 3.4. Compressive and Flexural Strength of AAC Mortars

Table 4 presents the effects of incorporating CH in the activator solution on the compressive and flexural strength of AAC mortars. Since there was no strength development in the Mix 1 samples at ages of 2 and 7 days, the compressive and flexural strengths are shown as zero for these mixtures in Table 4. At an age of 28 days, the compressive strength of Mix 1 (SS5%) reached 26.0 MPa, which was significantly lower than the strength levels obtained by using sodium hydroxide or silicate activators in other studies [23,24]. It can be seen from the Table 4 that a 1% CH addition (Mix 3) can improve the reaction rate at the early ages, as the compressive strength reached 12.3 and 31.3 MPa at ages 2 and 7 days, respectively. After 28 days of curing, Mix 3 showed a compressive strength of 45.2 MPa, which was significantly higher than Mix 1. A 0.5% CH addition (Mix 2) also provided an equivalent compressive strength to Mix 3 at later stage (28 days), but with significantly lower early strength (2 days). Conversely, with higher CH addition of 2.5% (Mix 4) showed appropriate compressive strength at the early ages but significantly lower compressive strength as compared to other three mixtures. In conclusion, choosing an appropriate addition of CH (1%) can increase the compressive strength at both early and later ages.

The flexural strength of AAC mortars exhibited the same tendency as the compressive strength. Flexural strength is more sensitive to cracks than compressive strength [25]. An incorporation of CH into the sodium sulfate activator can significantly increase the flexural strength of AAC mortars at the early ages, which is also consistent with the MIP and microstructure results that will be presented in Section 3.5 and Section 3.6, showing lower porosity and fewer cracks for the mixtures containing CH. Earlier studies [26,27,28] pointed out that the incorporation of Ca(OH)_2_ into the Na_2_SO_4_-activated GGBFS mixtures can enhance the strength development. Shi et al. [29] reported that the Na_2_SO_4_ induced the formation of ettringite, which was beneficial to early strength. Jeong et al. [30] observed that excessive addition of Ca(OH)_2_ (6.25%) did not improve the 28-day strength significantly. This finding agrees with the current work where the addition of 2.5% Ca(OH)_2_ did not increase the mechanical performance at later ages.

As compared to Portland cement, the sodium sulfate-activated slag mixtures generally showed lower compressive strength [9]. Previous studies [31,32] reported that the pH value of the activator solution plays an important role in the reaction process and influences the nature of the reaction products of the alkali-activated slag systems. It was previously stated that when the pH of the activator solution is less than 9.5, the hydration of the slag cannot proceed. The pH of the activator solution should be higher than 11.5 in order to enable efficient hydration of the slag [31]. Lower pH of the sodium sulfate activator can cause longer setting times and lower mechanical properties due to slow reactions of the slag in a low-pH environment. As shown in this study, the addition of a small amount of CH can efficiently improve the pH value of the activator solution, thereby resulting in higher compressive strength.

### 3.5. Pore Structure of AAC Pastes

Table 5 presents the total porosity values of the samples at ages 2, 7, and 28 days for Mixtures 1 and 4, measured by MIP. It should be noted that the total porosity of Mix 1 at 2 and 7 days could not be measured by MIP as the samples had not hardened at these ages. Table 5 shows that the total porosity of the samples decreased over time due to the ongoing reactions of GGBFS. At the age of 2 days, sample SS5% + CH2.5% had the highest porosity of 28% and the porosity decreased with elapsed curing time as expected.

**Figure 8:**
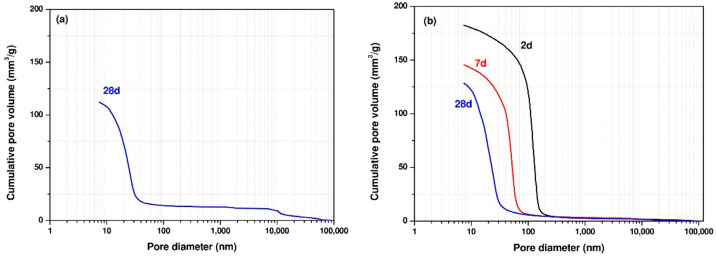
Pore size distributions determined by MIP (**a**) SS5%, (**b**) SS5% + CH2.5%.

Figure 8 presents the pore size distribution for Mixes 1 and 4 at the curing ages of 2, 7, and 28 days. As the curing age proceeded, the total porosity decreased and the pore size distribution curve shifted to smaller pore sizes for all samples, reflecting formation of a denser microstructure. It was clear that the reduced porosity and refined microstructure of samples at later ages was related to a higher reaction degree of the samples. As the reaction continued, more reaction products were formed, filling the pore spaces, and consequently leading to a denser microstructure. It could be seen that CH addition can significantly reduce the porosity at the early ages.

**Figure 9:**
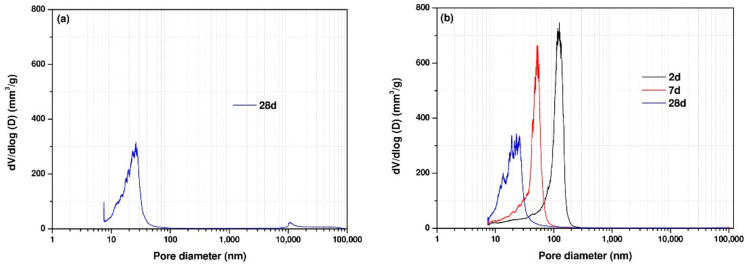
Differential curves from the particle size distribution curves (**a**) SS5%, (**b**) SS5% + CH2.5%.

Figure 9 shows the differential curves from the pore size distribution curves. The differential curve of MIP pore size distribution in Portland cement pastes has two peaks, showing two distinct pore systems [33,34]. The first peak has pore diameters ranging from 10 to 100 nm, which corresponds to the gel pore system’s threshold pore diameter. And the second peak has a pore diameter greater than 100 nm, corresponding to the capillary pore system’s threshold pore diameter. In this study, a peak in the pore size ranging from 10 to 100 nm corresponding to the gel pores was identified in the differential curves of MIP pore size distribution for almost all samples at all curing ages except the sample SS5% + CH2.5% at the age of 2 days showing a peak having a pore diameter greater than 100 nm, corresponding to the capillary pores. With elapsed curing time, the peak generally shifts to a smaller pore diameter with lower intensity. Previous studies [35] also reported that the peak with pore diameters greater 100 nm disappeared for the sodium hydroxide activated slag mixture with Na_2_O contents of 4%, 6% and 8%. The absence of the peak corresponding to capillary pores was primarily due to reaction products continuously growing into the pore space, resulting in a denser microstructure with decreased capillary porosity, smaller and only partially connected capillary pores. As a result, with time elapsing, the peak corresponding to capillary pores weakened and even disappeared. Previous studies [30] also reported that a significant strength development or dense microstructure formation could be achieved by incorporating CH into SS-activated GGBFS mixtures. Shi et al. [1] proposed that the SS induced the formation of ettringite, inducing strength development and dense microstructure formation. This can be explained that the porosity decreased at later stages possibly due to the formation of ettringite filling the pores.

### 3.6. Morphology of AAC Pastes

**Figure 10:**
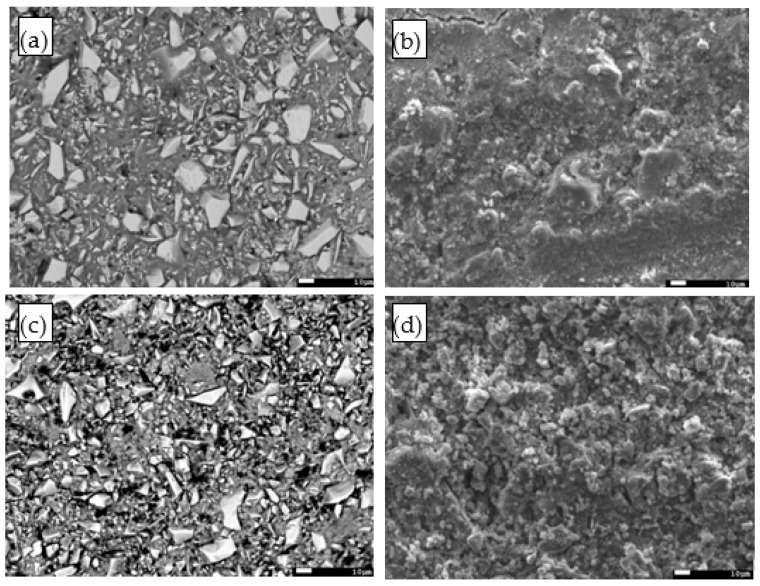
SEM images of AAC mixtures at the age of 28 days (**a**) BSE SS5%, (**b**) fractured sample SE SS5%, (**c**) BSE SS5% + CH2.5%, (**d**) fractured sample SE SS5% + CH2.5%.

Figure 10 shows the BSE-images and SE-images (fractural sample) of the AAC mixtures at 28 days. The gray areas in BSE images of Figure 10 denote the reaction products, the bright areas indicate the unreacted GGBFS grains, and the pores or cracks appears as the black areas. It is clear from Figure 10 that the reaction products surrounded the unreacted GGBFS grains for all samples for the sample SS5%, some cracks were shown in the image, and this was also consistent with its lower compressive strength and higher porosity determined by MIP. However, when introducing CH into SS activator solution, a porous microstructure was formed as shown in Figure 10c. In addition, the BSE-image showed that the GGBFS particles were not surrounded by a solid and dense matrix. As a result, the SS5% + CH2.5% sample had higher porosity and lower compressive strength at 28 days. Moreover, it was clear that the fractural surface of SS5% at the age of 28 days showed a better-packed microstructure compared to SS5% + CH2.5%.

Image analysis on BSE pictures was also conducted to predict the reaction degree of the AAC mixture described in Section 2.2 and Equation (2). As can be seen in Table 6, the reaction degree of SS5% + CH2.5% mixture at the age of 28 days reached approximately 60%, which was slightly lower than that of the sample SS5%.

It could be concluded that excessive CH addition can increase the early reaction rate but is not effective in improving the reaction rate at the later ages. According to previous findings by Fu et al. [13], CH would react with sodium sulfate, resulting in the formation of gypsum and NaOH, leading to a higher pH value of the suspension and effectively driving an accelerated GGBFS reaction. They stated that this mechanism appeared to make sense since the sufficient sulfate had a tendency to drive Ca removal from solution. This explains why CH can significantly increase the early reaction rate.

### 3.7. FTIR and XRD Analyses of AAC Pastes

**Figure 11:**
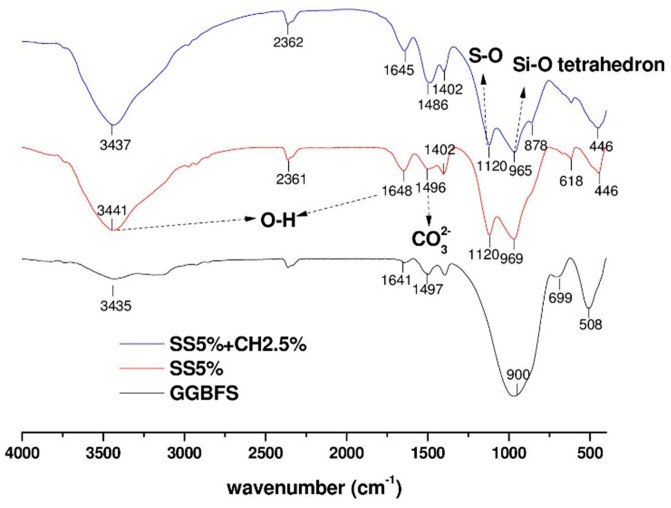
FTIR spectra of GGBFS and AAC pastes at the age 28 days.

**Figure 12:**
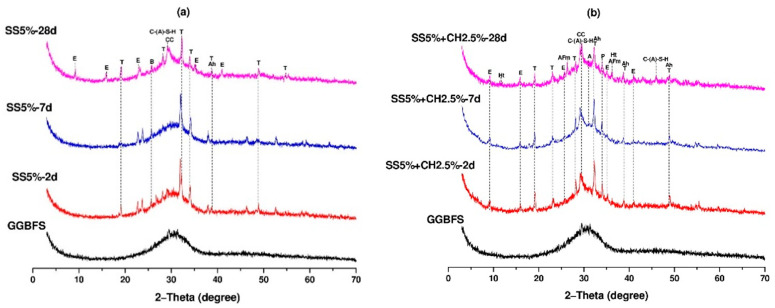
XRD spectra of AAC pastes (**a**) SS5%, (**b**) SS5% + CH2.5% (E: ettringite, T: thenardite, B: bassanite, CC: calcite, C-(A)-S-H: calcium-alumniosilicate-hydrate, Ah: anhydrite, Ht: hydrotalcite, AFm: a monosulfate phase, A: akermanite, and P: portlandite).

FTIR and XRD were performed on the samples at the age of 28 days. The FTIR spectra of AAC pastes activated by different activator solutions are shown in Figure 11. For precursor material GGBFS, the bands at around 1641 and 3435 cm^−1^ were assigned to the bending and stretching vibration of bound water molecules, respectively [36,37]. The band centered at 900 cm^−1^ was associated with the asymmetric stretching vibration mode of Si-O-T bonds (T: tetrahedral Si or Al) [38]. An increase in the wavenumber of this band is linked to higher degrees of crosslinking of silicates and formation of a silicate with a very low concentration of calcium [38]. Additionally, the bands centered at 1497 cm^−1^ were attributed to the stretching vibration of O-C-O bonds of ${\mathrm{CO}}_{3}^{2-}$ groups [38,39]. All samples showed OH groups at similar locations of absorption bands in general, which were at around 1641–1650 cm^−1^ and 3435–3449 cm^−1^, indicating the presence of chemically bound water within the reaction products [40]. Small bands at around 1486, 1496, and 1497 cm^−1^ were observed in the samples SS5% + CH2.5%, SS5% and the GGBFS precursor, respectively. The FTIR peak at around 1490 cm^−1^ is associated with vaterite as a result of carbonation [41]. However, its amount seems too low to be detectable in the XRD analysis (Figure 12). The main absorption band at around 965 cm^−1^ for all samples used in this study was assigned to the asymmetric stretching vibration of Si-O terminal bonds [42]. This is the representative vibration band of AAC that indicates the formation of C-A-S-H type gels with short chain structures. As can be seen in Figure 11, the Si-O bond was originally located at around 900 cm^−1^ in the unreacted slag, while for samples SS5% and SS5% + CH2.5% the bands at around 965 and 969 cm^−1^, respectively became narrower and shifted to higher wavenumbers than in the original slag, indicating the formation of a more polymerized Si-O network. Additionally, in the SS5% and SS5% + CH2.5% samples, a new peak was found near 1120 cm^−1^ when compared to the original slag. This peak is associated with the S-O bond stretching [42,43,44], indicating the ettringite formation.

The XRD diffractograms of AAC pastes activated by SS5% and SS5% + CH2.5% are shown in Figure 12. For SS5% ettringite, C-(A)-S-H, calcite, anhydrite, bassanite, and thenardite peaks were observed in the 28-day samples. At 2 and 7 days there was no C-(A)-S-H peak however, a limited ettringite peak was observed. The apparent ettringite and C-(A)-S-H peaks observed at the age of 28 days indicate the increased reaction rate by time. These observations agree well with the mechanical test results given in Table 4. For SS5% + CH2.5% activator case, C-(A)-S-H, ettringite, monosulfate, hydrotalcite, calcite, anhydrite, portlandite, and thenardite peaks were observed at the age of 28 days. The ettringite and C-(A)-S-H peaks were apparent from the age of 2 days (Figure 12b) indicating the rapid increase in reaction. The presence of portlandite showed that not all of the Ca(OH)_2_ was participated to the reaction. This may be the reason for the slightly lower mechanical performance of the SS5% + CH2.5% sample compared to SS5%.

## 4. Conclusions

This study investigated the effect of Ca(OH)_2_ addition into the sodium sulfate activator solution on the fresh state properties, mechanical properties, pore structure and morphology of AAC. The following conclusions can be drawn from this study:

- The AAC mixture activated by the sole sodium sulfate activator solution could not develop strength until the age of 7 days. Early age strength development of sodium sulfate activated slag mixtures could be improved by Ca(OH)_2_ addition to the activator solution.
- The very long setting times of the sole sodium sulfate activated AAC mixture were significantly shortened by the addition of Ca(OH)_2_. The yield stress and plastic viscosity could also be significantly improved by the addition of an appropriate amount of Ca(OH)_2_.
- The binary usage of Ca(OH)_2_ and sodium sulfate activators exhibited a higher reaction degree at the early ages compared with sole usage of sodium sulfate activator. However, the addition of Ca(OH)_2_ higher than 1% led to a more porous microstructure at later ages. The porosity of the mixture with the 2.5% Ca(OH)_2_ addition at later ages was found to be slightly higher than the mixture activated by only sodium sulfate activator.
- The FTIR and XRD analyses confirmed that the main reaction products in sodium sulfate activated AAC mixtures with or without Ca(OH)_2_ were C-A-S-H and ettringite.

## Figures and Tables

**Table 1:** Chemical composition of the GGBFS used in the study.

Precursor	CaO	SiO_2_	Al_2_O_3_	MgO	SO_3_	TiO_2_	K_2_O	Na_2_O	Fe_2_O_3_	MnO	Others
GGBFS	40.8	33.3	12.3	7.84	2.30	1.29	0.67	0.44	0.39	0.36	0.31

**Table 2:** Mixture design of alkali-activated slag mixtures.

Mix	Mixture Notation	W/SB *	Na_2_O % **	Ca(OH)_2_ % **
1	SS5% ***	0.42	5	0
2	SS5% + CH0.5%	0.42	5	0.5
3	SS5% + CH1%	0.42	5	1.0
4	SS5% + CH2.5%	0.42	5	2.5

* The sum of GGBFS and dry part of activator solution are considered as solid binder (SB). ** By mass of GGBFS content. *** SS and CH indicate Na_2_SO_4_ and Ca(OH)_2_.

**Table 3:** Initial and final setting times of AAC pastes.

Mixture	Mixture Notation	Initial Setting Time (min)	Final Setting Time (min)
1	SS5%	2940	3960
2	SS5% + CH0.5%	402	1251
3	SS5% + CH1.0%	330	738
4	SS5% + CH2.5%	396	990

**Table 4:** Flexural and compressive strength of AAC mixtures.

Mixture	Mixture Notation	Flexural Strength (MPa)			Compressive Strength (MPa)		
		2-Day	7-Day	28-Day	2-Day	7-Day	28-Day
1	SS5%	0	0	4.9 ± 0.4	0	0	26.0 ± 0.5
2	SS5% + CH0.5%	1.0 ± 0.4	5.0 ± 0.4	6.8 ± 0.3	3.7 ± 0.4	23.2 ± 0.6	45.2 ± 1.3
3	SS5% + CH1.0%	4.1 ± 0.2	7.3 ± 0.2	8.6 ± 0.5	12.3 ± 0.3	31.3 ± 0.8	45.2 ± 2.3
4	SS5% + CH2.5%	4.0 ± 0.1	6.6 ± 0.1	7.2 ± 0.2	9.7 ± 0.2	17.3 ± 0.2	23.4 ± 0.1

**Table 5:** The total porosity of AAC mixtures.

Mixture	Mixture Notation	2 d Porosity (%)	7 d Porosity (%)	28 d Porosity (%)
1	SS5%	not hardened	not hardened	19.8
4	SS5% + CH2.5%	28.0	23.5	20.2

**Table 6:** Reaction degree of AAC pastes.

Mixture	Mixture Notation	2 d Reaction Degree (%)	7 d Reaction Degree (%)	28 d Reaction Degree (%)
1	SS5%	not hardened	not hardened	61.5 ± 0.2
4	SS5% + CH2.5%	47.8 ± 1.2	52.5 ± 0.5	60.2 ± 1.0
